# Elevated C-reactive protein is associated with disease progression in patients with mild Crohn’s disease

**DOI:** 10.1186/s40064-016-2606-6

**Published:** 2016-06-24

**Authors:** Min Seob Kwak, Kyung-Jo Kim, Sang Hyoung Park, Dong-Hoon Yang, Byong Duk Ye, Jeong-Sik Byeon, Seung-Jae Myung, Suk-Kyun Yang

**Affiliations:** Department of Gastroenterology, Asan Medical Center, University of Ulsan College of Medicine, 88, Olympic-Ro 43-Gil, Songpa-gu, Seoul, 05505 Republic of Korea

**Keywords:** Crohn’s disease, Azathioprine, 6-mercaptopurine

## Abstract

**Background:**

Few studies have been conducted on the progression of mild Crohn’s disease (CD). We aimed to investigate the natural course in mild CD patients with or without bowel damage, to identify predictors of bowel resection and to calculate the requirement for rescue medication.

**Methods:**

A total of 104 patients with mild activity (150 < CDAI < 220) with or without bowel damage were identified from among 1050 CD patients between January 2008 and May 2014. Univariate and multivariate analysis was used to identify factors associated with bowel resection. The cumulative probabilities of bowel resection and rescue medication such as steroids or anti-TNF agents were calculated.

**Results:**

The median follow-up duration was 28.2 months (IQR 26.7). Cumulative probabilities of bowel resection were 0.2, 11.8 and 42.4 % at 1, 3 and 5 years respectively. The CD patients with bowel damage had a higher bowel resection rate than those without bowel damage (*P* < 0.001). The cumulative probabilities of corticosteroid-requirement were 3.0, 19.6 and 78.4 % of patients at 1, 3 and 5 years, respectively, and 2.1, 11.9 and 56.1 %, in terms of the cumulative probabilities of requiring anti-TNF agents. Patients with elevated CRP (>1.6 mg/dL) were more likely to undergo bowel resection (*P* = 0.032).

**Conclusions:**

Even in CD patients with mild disease activity, the cumulative probability of bowel resection is not low if they have bowel damage or elevated CRP at baseline. Mild CD patients with bowel damage or elevated CRP at baseline need special attention.

## Background

Crohn’s disease (CD) is a chronic persistent inflammation of the gastrointestinal tract of unclear etiology; with time it leads to bowel damage (Abraham and Cho 2009).

Global therapeutic strategies for CD patients involve step-up or top-down approaches. The step-up strategy is adopted individually on a sequential basis. The top-down strategy involves the early introduction of immunosuppressive and biological treatments to halt the progressive bowel damage that occurs during the course of the disease in patients with moderate to severe activity (Beaugerie et al. 2006; D’Haens et al. 2008; Dignass et al. 2010). However, an appreciable proportion of patients with CD have a mild pattern of disease for which budesonide and mesalazine remain the preferred treatment (Dignass et al. 2010). The variable clinical spectrum makes it difficult to predict outcomes in mild patients with CD (Peyrin-Biroulet et al. 2010b). A better understanding of the natural course of CD patients with mild disease activity may be helpful in improving their management.

Little information is available on the progression of mild CD and the factors associated with long-term mild IBD (Cosnes et al. 2012; Reenaers et al. 2016). This study was conducted to investigate the course of mild CD in a hospital-based cohort. In addition, we identified risk factors for long-term outcomes such as bowel resection, and requirements for rescue medication.

## Methods

This study was approved by the Institutional Review Board of Asan Medical Center (IRB No 2015-0327) and informed consent was waived for this retrospective study.

### Study population

This study was performed retrospectively. The patients with CD were identified at the Asan Medical Center, a tertiary university hospital in Seoul, South Korea, between January 2008 and May 2014. CD diagnosis was based on conventional clinical, radiologic, endoscopic, and histopathologic criteria (Lennard-Jones 1989). We identified not only newly-diagnosed mild CD patients but also those referred from other hospitals after diagnosis. Mild activity was defined by the Crohn’s Disease Activity Index (CDAI) score (150 < CDAI < 220) with no documented history of use of corticosteroids or anti-TNF agents or bowel surgery at the initial clinic visit. The referred patients we required to have mild activity at the time of referral and no history of use of steroids, immunomodulators or anti-TNF agents. For the patients referred after the diagnosis of CD, prior medical information was obtained retrospectively by reviewing the medical records provided, or by interviewing the patients at their first visit. A minimum follow-up of 6 months was required for inclusion. The diagnosis was systematically re-evaluated at each scheduled visit.

### Description of variables

Information on age, gender, date of CD diagnosis, time between onset of symptoms and diagnosis, follow-up duration, date of surgery such as intestinal resection, date of disease-related hospitalization, disease location and disease behavior were retrospectively extracted from the medical records. Disease location and behavior were defined according to the Montreal classification (Satsangi et al. 2006). Bowel damage was defined as the presence of strictures, fistula or abscess on endoscopic or radiologic examination. We defined the stricture as luminal narrowing with prestenotic dilatation or with/without obstructive symptoms in small bowel or colon (Peyrin-Biroulet et al. 2010a).

### Treatment strategies and follow-up protocol

Our strategies for treating CD were based on a step-up approach, with addition of more potent therapies if and when patients became unresponsive to first-line or less toxic agents. Oral aminosalicylates were used to induce and maintain remission in patients with mild to moderate active disease especially until the mid-2000s (Park et al. 2014). Azathioprine (AZA) or 6-mercaptopurine (6-MP) was used as maintenance therapy for steroid-dependent, steroid-refractory, or fistulizing patients in selected cases, mainly after resective surgery, but on a more widespread basis from mid to late 2000. To minimize the occurrence of unexpected severe leucopenia with AZA, we used the following strategy. AZA was started at 25–50 mg/day, and increased by 25–50 mg/day every 2–4 weeks to a maximum dose of 2.0–2.5 mg/kg/day while leukocyte levels were being monitored. If a rapid decrease in the leukocyte count or leucopenia occurred, the AZA therapy dose was decreased or the therapy was stopped for a while and restarted at a lower dose. The dose of AZA was defined as the maintenance dose that induced remission.

The 6-MP dose was converted to an equivalent AZA dose by multiplying by 2.08 (Sandborn 2001). Short-term systemic corticosteroid therapy (oral prednisone 40–60 mg/day) was used to treat patients with moderate to severe active disease or those who did not respond to oral aminosalicylates, then tapered and discontinued over 2–3 months. Anti-TNF agents were administered to patients with moderate to severe active disease who were unresponsive to therapy with corticosteroids and/or AZA/6-MP from the early-2000s. Surgical procedures including bowel resection, strictureplasty, or drainage of abscesses were performed to treat medically refractory disease or complications such as intestinal obstruction, perforation, abscess, or fistula (Hanauer 2003). The laboratory test including erythrocyte sedimentation rate (ESR), C-reactive protein (CRP), hematocrit, albumin, and CDAI (Best et al. 1979) were measured from the first visit after referral or from the time of diagnosis regularly during the follow-up period in all the patients.

### Primary and secondary endpoint measures

The primary endpoint was the cumulative probability of bowel resection. Non-resectional surgery such as seton insertion, fistula repair, perianal surgery, or abscess drainage was excluded. The secondary endpoints were assessed between the groups as follows: cumulative probabilities of (1) steroid requirement, (2) anti-TNF agents requirement, and (3) disease-related hospitalization. Furthermore, the cumulative probabilities of the primary and secondary endpoints were compared in the patients with bowel damage and those without bowel damage.

### Statistical analysis

Statistical analyses were performed with SPSS software (version 21.0 for Windows; SPSS, Inc., Chicago, IL, USA). Continuous variables were compared using the two-tailed Student’s t test, and categorical data were compared using a two-tailed χ^2^ test or Fisher’ exact test. Rates of corticosteroid requirement, anti-TNF agents requirement, hospitalization, and resectional surgery after treatment were analyzed using Kaplan–Meier survival curves for the grouped factors over the intervention period. We analyzed factors associated with surgical resection by logistic regression analysis. *P* values <0.05 were considered to indicate statistical significance. All variables with *P* values of <0.1 were included in the regression analysis.

## Results

### Baseline characteristics

**Fig. 1:**
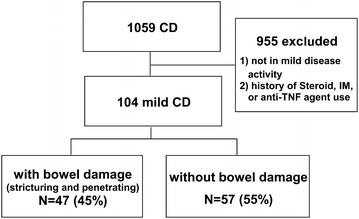
Flow diagram of the patient selection process

Figure 1 is a flow chart of the patient selection pathway. A total of 104 CD patients with mild disease activity were identified, and were classified into those with bowel damage (n = 47) and those without bowel damage (n = 57); their baseline characteristics are listed in Table 1. Age at diagnosis was higher in the patients with bowel damage than in those without bowel damage. The duration of symptoms before diagnosis was longer in the former group (36.0 vs. 11.1 months; *P* < 0.001). The cut-off point above which levels of CRP were considered high was 1.6 mg/dL, which was the median value of CRP in these patients. Eighteen out of 19 patients with stenosis showed low CRP level (≤1.6 mg/dL) at baseline. Ten out of 28 patients with penetrating complications showed low CRP level at baseline.

**Table 1:** Clinical characteristics of mild CD patients according to presence of bowel damage

Characteristic	Total (n = 104)	Patients with bowel damage (n = 47)	Patients without bowel damage (n = 57)	*P* value
Age at diagnosis, year, median (IQR)	28.0 (11.8)	32.0 (13.0)	24.0 (9.0)	0.047
Gender, (%)				0.067
Male	85 (81.7)	42 (89.4)	43 (75.4)	
Female	19 (18.3)	5 (8.6)	14 (24.6)	
Symptom duration before diagnosis, month, median (IQR)	15.9 (40.1)	36.0 (66.9)	11.1 (22.2)	<0.001
Location, (%)				0.431
L1 (ileal)	20 (19.2)	12 (25.5)	8 (14.0)	
L2 (colonic)	4 (3.8)	1 (2.1)	3 (5.3)	
L3 (ileocolonic)	45 (43.3)	19 (40.4)	26 (45.6)	
L4 (only upper GI)	0 (0.0)	0 (0.0)	0 (0.0)	
L1–L4	11 (10.6)	5 (10.6)	6 (10.5)	
L2–L4	0 (0.0)	0 (0.0)	0 (0.0)	
L3–L4	24 (23.1)	10 (21.3)	14 (24.6)	
Behavior at diagnosis, (%)				0.002
B1 (non-stricturing, non-penetrating)	23 (22.1)	0 (0.0)	23 (22.1)	
B2 (stricturing)	11 (10.6)	11 (10.6)	0 (0.0)	
B3 (penetrating)	18 (17.3)	18 (17.3)	0 (0.0)	
B1-P	34 (32.7)	0 (0.0)	34 (32.7)	
B2-P	8 (7.7)	8 (7.7)	0 (0.0)	
B3-P	10 (9.6)	10 (9.6)	0 (0.0)	
BMI, median (IQR)	19.7 (3.7)	19.2 (3.9)	20.3 (3.6)	0.206
CRP, median (IQR)	1.6 (3.6)	1.6 (3.6)	1.6 (3.6)	0.268
ESR, median (IQR)	38.0 (50.5)	32.0 (49.0)	30.0 (58.0)	0.981
Hct, median (IQR)	39.5 (6.9)	39.1 (7.0)	39.9 (6.9)	0.800
CDAI, median (IQR)	180.4 (36.6)	187.4 (40.2)	177.6 (36.3)	0.975

*IQR* inter-quartile range, *ASA* aminosalicylic acid, *BMI* body mass index, *CRP* C-reactive protein, *ESR* erythrocyte sedimentation rate, *Hct* hematocrit, *CDAI* Crohn’s disease activity index, *NA* not applicable

### Primary and secondary endpoints

**Fig. 2:**
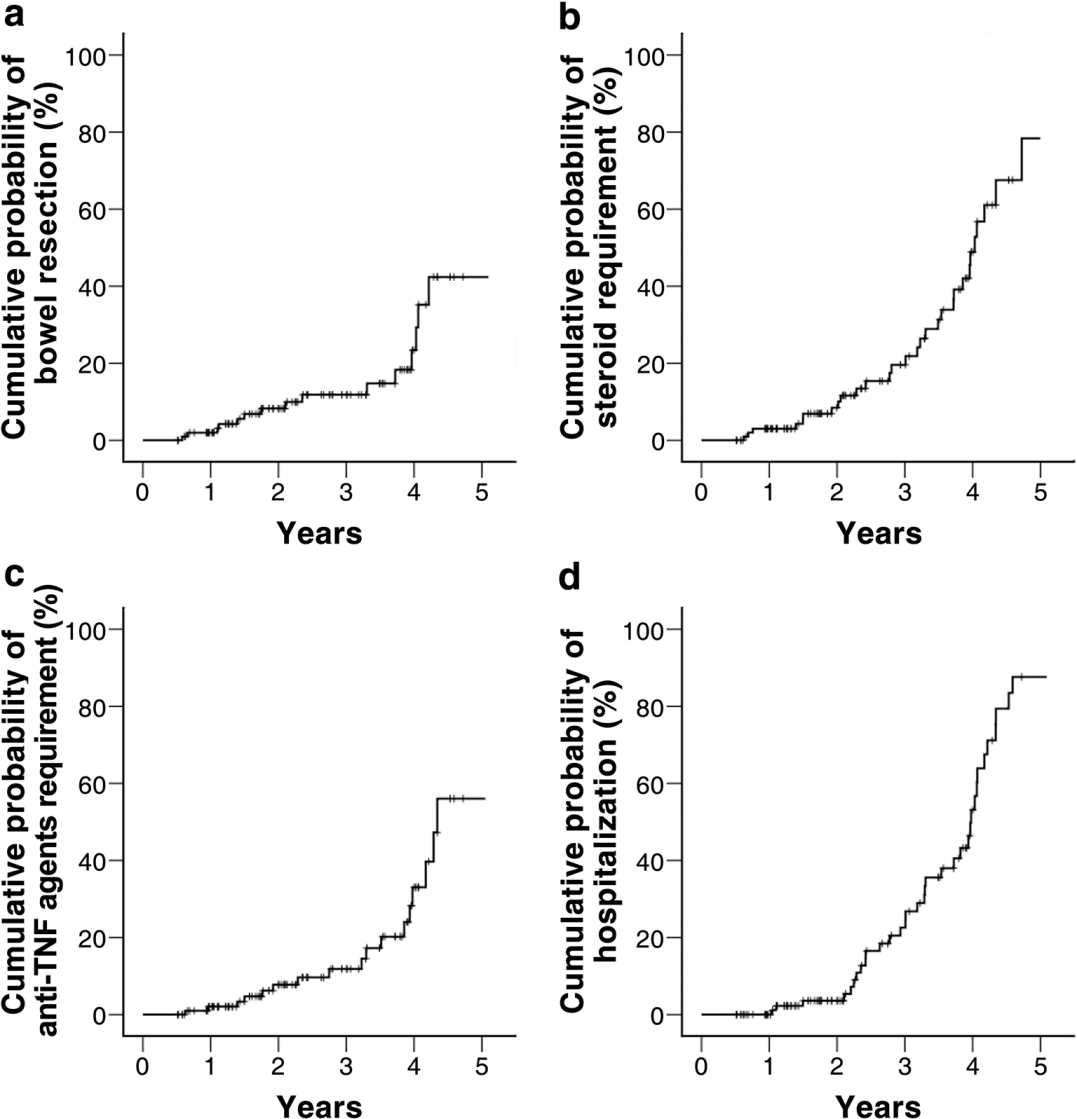
The cumulative probabilities of bowel resection (**a**), steroid requirement (**b**), anti-TNF agent requirement (**c**), and hospitalization (**d**) in patients with mild activity

The median follow-up duration was 28.2 months (range 64.2 or IQR 26.7). Additional rescue therapy was also administrated in patients [17 (36 %) with bowel damage, 21 (37 %) without bowel damage, respectively] during their disease course except emergency cases. Unfortunately, the number of patients is too small to measure the risk. The cumulative probabilities of bowel resection were 0.2, 11.8 and 42.4 % of patients, respectively, at 1, 3 and 5 years of treatment (Fig. 2a). The cumulative probabilities of corticosteroid-requirement were 3.0, 19.6 and 78.4 % of patients at 1, 3 and 5 years, respectively, (Fig. 2b) and in 2.1, 11.9 and 56.1 %, in terms of cumulative probabilities of anti-TNF agent requirement (Fig. 2c). The cumulative probabilities of disease-related hospitalization were 0.0, 22.6 and 87.6 % at 1, 3, at 5 years after the start of treatment (Fig. 2d).

### Analysis according to the presence of bowel damage at enrollment

**Fig. 3:**
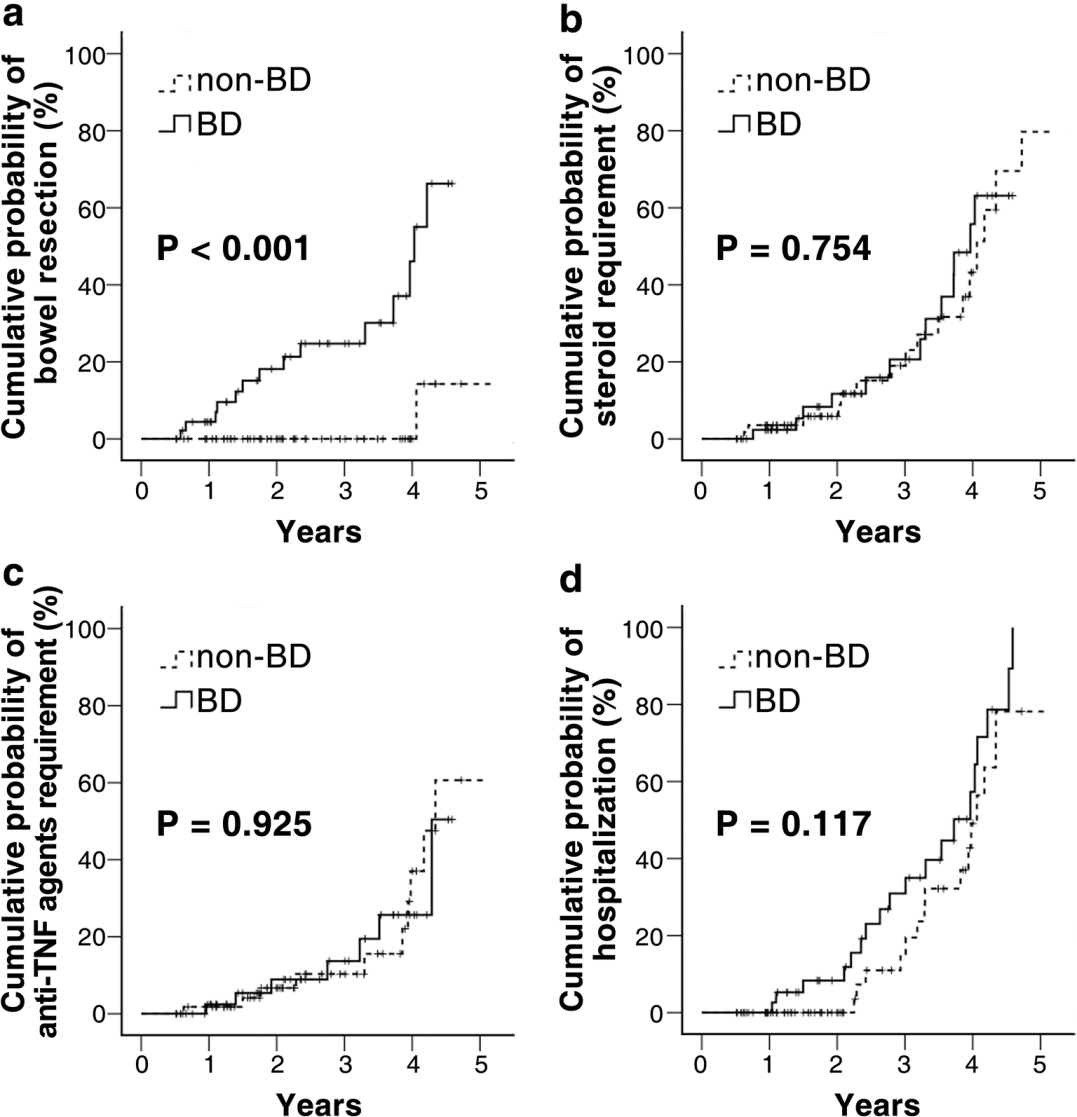
Cumulative probabilities of bowel resection (**a**), steroid requirement (**b**), anti-TNF agent requirement (**c**), and hospitalization (**d**) with and without bowel damage at diagnosis

There were no differences between the patients with bowel damage and those without bowel damage regarding the cumulative probabilities of corticosteroid-requirement (*P* = 0.754) (Fig. 3b), anti-TNF agent-requirement (*P* = 0.925) (Fig. 3c), and disease-related hospitalization (*P* = 0.117) (Fig. 3d). However, a higher proportion of those with bowel damage underwent bowel resection (*P* < 0.001) (Fig. 3a).

### Factors associated with intestinal resection

Bowel resection was performed in 23 of the 104 (22.1 %) patients during follow-up. The reasons for surgery were strictures (n = 5) and penetrating complications (n = 18). None of the patients with strictures underwent an endoscopic balloon dilatation. Perianal fistula was associated with bowel resection in univariate analysis (OR 3.724; 95 % confidence interval 1.330–10.423; *P* = 0.012) and in multivariate analysis (OR 3.885; 95 % confidence interval 1.189–12.691; *P* = 0.025) (Table 2). In addition, there were significant associations between bowel resection and male gender in multivariate analysis (OR 9.342; 95 % confidence interval 1.028–84.895; *P* = 0.047) (Table 2). CRP at baseline was associated with bowel resection in univariate (OR 1.133; 95 % confidence interval 1.009–1.271; *P* = 0.034) (Table 2) and multivariate analysis (OR 1.196; 95 % confidence interval 1.046–1.368; *P* = 0.009) (Table 2). However, age, ESR, hematocrit, and body mass index (BMI) were not significant predictors of bowel resection in univariate analysis (Table 2).

**Table 2:** Univariate and multivariate analysis of factors at diagnosis related to subsequent surgical resection

Variable	OR (95 % CI)	*P* value
Univariate		
Age	1.060 (1.993–1.093)	0.097
Gender		
Male	6.286 (0.792–49.880)	0.082
Female		
ESR	1.002 (0.988–1.017)	0.780
CRP	1.133 (1.009–1.271)	0.034
Hct	0.968 (0.892–1.051)	0.437
BMI	0.959 (0.825–1.115)	0.584
Perianal fistula		
Yes	3.724 (1.330–10.423)	0.012
Multivariate		
Age	1.037 (0.980–1.098)	0.205
Gender		
Male	9.342 (1.028–84.895)	0.047
Female		
CRP	1.196 (1.046–1.368)	0.009
Perianal fistula		
Yes	3.885 (1.189–12.691)	0.025

*ESR* erythrocyte sedimentation rate, *CRP* C-reactive protein, *Hct* hematocrit, *BMI* body mass index, *CDAI* Crohn’s disease activity index

**Fig. 4:**
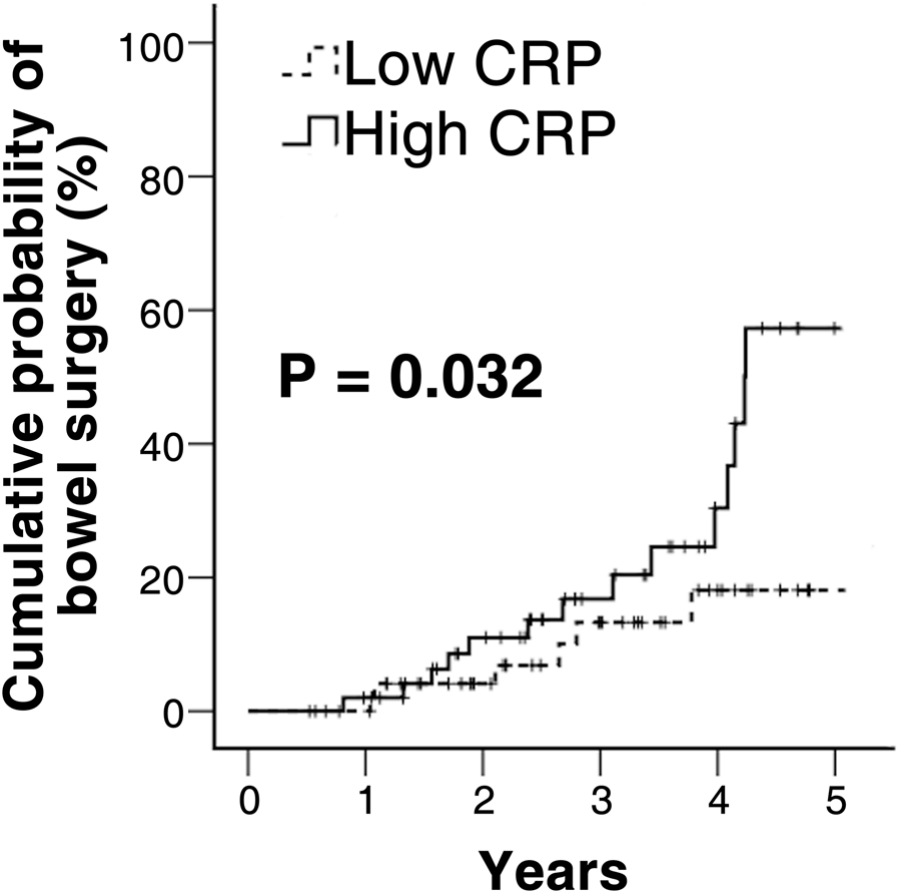
Kaplan–Meier bowel resection-free survival curves in patients with mild CD, stratified by low (n = 52) and high (n = 52) CRP level

Stratified analysis by level of CRP elevation demonstrated increased bowel resection in those with higher CRP (Fig. 4).

## Discussion

In our study about one-fifth of patients with mild activity underwent bowel resection during follow-up. Three-quarters of the patients required corticosteroids during their illness, and more than half required anti-TNF agents. In addition elevated CRP at baseline was predictive of intestinal resection.

Cohort studies indicated that more than half of the patients with CD between 1990 and 1994 had a mild course of disease during their first 10 years (Froslie et al. 2007). However, in our study only one-tenth of all CD patients had mild CD. The referral bias may contribute to the relatively small percentage of mild CD. However, we defined mild CD patients with both CDAI and history of medication use. We do not think the possibility of misclassification of CD patients with mild activity is high.

As far as we know, only one study has described the evolution of mild IBD; it analyzed the clinical significance and factors associated with inflammatory bowel disease that was considered to be mild 1 year after diagnosis (Reenaers et al. 2016). In that study, 90 % of CD patients lost their mild disease status over time (Reenaers et al. 2016).

However, our study differed from that study (Reenaers et al. 2016). First, we focused on CD and we assessed disease activity regularly, using the CDAI, which is a well-established research tool. As most IBD referral centers in Korea have their own outpatient clinic, assessing CDAI scores regularly is not difficult. Second, the former study defined mild as inflammatory luminal CD without bowel damage. We defined mild CD using “regardless of the presence of bowel damage” and found that about half of the patients with mild activity had bowel damage at baseline. We evaluated the small bowel and colon at the time of the first clinic visit in all patients, using ileocolonoscopy and CT enterography. Despite demonstrating objective evidence of bowel damage, about a half of the CD patients had mild disease activity. Those with bowel damage had a longer duration of symptoms before diagnosis and more often underwent intestinal resectional surgery during their disease course. This implies that prolonged symptom duration in mild patients with bowel damage habituates them to inflammatory signals. These findings have also been reported in a study of asymptomatic CD patients (Click et al. 2015).

The overall cumulative probability of bowel resection was not low in this study, which can be explained by the enrollment of mild CD patients with bowel damage at baseline. Of our patients, 2 % had undergone surgical resection at 1 year, and 21.8 and 42.4 % at 3 and 5 years, respectively (Fig. 2a). Our surgical resection rates differed little from the results of the above study (Click et al. 2015) despite the mild clinical activity at diagnosis. Bowel resection rates in CD patients ranged from 7 to 59 % at 5 years in a recent review (Wolters et al. 2004; Chatu et al. 2014). In the present study, one-third of the patients received immunomodulators regardless of bowel damage after enrollment.

The cumulative rates of introduction of anti-TNF agents were 2.1 and 56.1 % at 1 and 5 years, respectively (Fig. 2c), lower than the 23.2 and 67.0 % in a previous study (Peyrin-Biroulet et al. 2011). These findings are quite surprising in that anti-TNF agents can be indicated in even in mild CD at baseline.

It is important to identify predictive factors at the time of diagnosis, and more intensive intervention may be recommended in selected CD patients with poor prognostic factors.

Beaugerie et al. (2006) reported that age less than 40 years, the presence of perianal disease, and the need for steroid treatment at diagnosis were predicted disabling course of CD over subsequent 5 years. C-reactive protein (CRP) was only biological parameter to predict a more severe clinical course of CD (Henriksen et al. 2008). In this study, most of the identified patients (82.7 %, 86/104) were less than 40 years. We excluded the steroid users or immunosuppressant users from the cohort initially. Thus, we only added the presence of perianal fistula in the univariate and multivariate analysis. (Beaugerie et al. 2006; Henriksen et al. 2008; Cerqueira and Lago 2013).

We confirmed that patients with bowel damage at diagnosis had a higher bowel resection rate than those without bowel damage. In several studies, bowel damage, such as stricturing or penetrating behavior, was associated with increased risk of surgical recurrence (Fowler et al. 2014; Sjoberg et al. 2014; Moon et al. 2014).

We demonstrated that patients with elevated baseline CRP levels had a higher bowel resection rate even in patients with mild activity. It is well-known that elevated CRP are regarded as indicators of disease activity and the predictors of the risk of relapse in patients with CD who are in clinical remission (Consigny et al. 2006; Desai et al. 2007; Koelewijn et al. 2008; Reinisch et al. 2012). Moreover, in a subgroup analysis, a significant association was found between CRP level at first visit and subsequent surgical resection (Henriksen et al. 2008). Some argues that CRP levels may be confounders with bowel damage in this study. However, Henriksen reported that no differences in CRP levels were observed between subgroups of patients categorised according to the Vienna classification related to behaviour (B1–B3) of the disease, although C-reactive protein (CRP) was only biological parameter to predict a more severe clinical course of CD (Henriksen et al. 2008). In our study, baseline CRP level was not different between the patients with bowel damage and those without bowel damage. Thus, bowel damage was not included into multivariate analysis.

An additional finding was that male gender was associated with bowel resection unlike in a previous study (Polito et al. 1996). We do not know why male gender is associated with bowel resection. However, Korean CD patients have a male predominance (Park et al. 2014). Young female Korean patients are usually reluctant to undergo bowel resection scar. These reasons probably contribute to the gender difference in bowel resection rate.

The major limitation of our study is that it was a retrospective, observational study, which had potential for referral bias and confounding factors. Secondly, although we aimed to investigate long-term outcomes, the length of follow-up achieved was not adequate. Third, we did not examine serologic reactivity to bacterial antigens, which are well-known predictors of fibrostenosing behavior in Western populations (Dubinsky et al. 2006). However, more time will be needed to incorporate serological tests into clinical practice because of their high cost in Korea. Forth, the data on smoking history, endoscopic and radiologic findings other than the presence of bowel damage was not complete because of the retrospective nature of the study. Another limitation comes from the small size of the studied population; further prospective studies with larger numbers of patients are needed. Despite of the several limitations, our study may provide useful insight into the disease course in mild CD patients, given the paucity of knowledge of this matter.

## Conclusions

In conclusion, we found that mild CD patients with bowel damage at initial evaluation and with elevated CRP may have worse long-term outcomes than those without bowel damage and with normal CRP levels. Attention should be paid to such patients, who may need a different therapeutic strategy despite their mild disease activity. We suggest that there may be a role for early intervention in mild CD.
